# Case Report: Near-fatal adverse effects of dopamine agonist for the treatment of restless legs syndrome

**DOI:** 10.3389/fphar.2025.1697935

**Published:** 2025-10-31

**Authors:** Jeremiah B. Joyce, Liliana Patarroyo Rodriquez, Christopher L. Sola

**Affiliations:** Department of Psychiatry and Psychology, Mayo Clinic, Rochester, MN, United States

**Keywords:** dopamine agonist, restless legs syndrome, chronic pain, adverse effects, suicidal ideation

## Abstract

**Background:**

The dopaminergic system plays a central role in motor control, goal-directed behavior, and the pathophysiology of multiple diseases including restless legs syndrome. Dopamine agonists such as pramipexole are commonly prescribed for the treatment of restless legs syndrome; however, growing recognition of potential serious adverse effects has led to updated clinical guidelines which now recommend against the routine use of dopamine agonists as initial therapy.

**Case Presentation:**

We present the case of a 64-year-old woman with severe restless legs syndrome and medically unexplained chronic pain who was psychiatrically hospitalized for active suicidal ideation. During her admission, it was discovered that a supratherapeutic dose of pramipexole was the source of iatrogenic harm. Her condition improved following medication taper, appropriate management of restless legs syndrome, and acute psychiatric interventions.

**Conclusion:**

Clinicians should be aware of the potential adverse effects associated with dopamine agonists and adhere to updated recommendations for the management of restless legs syndrome.

## 1 Introduction

Restless legs syndrome (RLS) is a sleep-related, sensorimotor disorder characterized by an irresistible urge to move the lower extremities, accompanied by uncomfortable sensations that worsen during rest, particularly in the evening, and are relieved by movement (Allen et al., 2014). Along with brain iron dysfunction, dopamine dysregulation is implicated in the pathophysiology of RLS. Given this connection, dopamine agonists (DAs) such as pramipexole are widely prescribed, with nearly 60% of United States patients receiving this medication class (Winkelman, 2021). Until early 2025, DAs were recommended as first-line pharmacologic therapy for RLS.

However, a growing body of scientific evidence including case reports (Signorelli et al., 2013; Cardon-Dunbar et al., 2017; Umbreit et al., 2021) and post-marketing surveillance (Mu et al., 2025) have described a broad spectrum of adverse effects associated with DA usage across multiple indications, including RLS and Parkinson’s disease. DAs have been implicated in the development of hallucinations, impulse control disorders such as pathological gambling, and even paradoxical worsening of RLS symptoms. Due to these concerns, professional medical societies, including the American Academy of Sleep Medicine (AASM), have updated their clinical practice guidelines to advise against routine use of DAs as initial therapy (Winkelman et al., 2025). This is the first case report of DA-related adverse effects following major revisions to clinical RLS management guidelines, highlighting the need for continued clinician education and dissemination of updated best practices.

We present a case of severe dopamine agonist toxicity in a 64-year-old woman receiving long-standing, supratherapeutic doses of pramipexole for the treatment of RLS. This case presented simultaneous occurrence of multiple dopaminergic adverse effects, including tactile and auditory hallucinations, compulsive skin excoriation, and an impulse control disorder characterized by financial exploitation. These iatrogenic complications culminated in active suicidal ideation and required psychiatric hospitalization with intensive therapeutic interventions. The case underscores the potential for medication-induced neuropsychiatric and behavioral manifestations to go unrecognized, leading to misdiagnosis and non-targeted interventions. Additionally, the case documents complete resolution of psychiatric and dermatological symptoms following pramipexole discontinuation, highlighting the reversibility of even severe and longstanding DA-related adverse effects.

## 2 Case description

A 64-year-old woman with treatment-resistant major depressive disorder presented to the emergency department in spring 2025 with acute exacerbation of chronic orofacial pain and active suicidal ideation. Her medical history included RLS, insomnia, chronic pain syndrome, type 2 diabetes mellitus complicated by diabetic peripheral neuropathy, a remote history of endometrial adenocarcinoma, and chronic migraine cephalgia. She reported financial stressors such as a recent loss of $6,000 through an online romance scam. Physical examination revealed multiple excoriated and encrusted lesions across the chin and lower face, which she compulsively manipulated while reporting paradoxical relief. She was alert and oriented, with a dysphoric affect, and endorsed active suicidal ideation with a specific plan to end her life via self-inflicted gunshot but instead sought emergency medical care. She was admitted to the inpatient psychiatry hospital for further evaluation and management.

Given the patient’s reports of chronic nocturnal restlessness and poor sleep, a medical workup was obtained and demonstrated iron deficiency anemia (IDA) with a hemoglobin of 9.1 g/dL, ferritin of 25 ng/mL, transferrin saturation (TSAT) of 4%, serum iron of 19 mcg/dL, total iron-binding capacity (TIBC) of 438 mcg/dL. Her psychopharmacological regimen was complex, consisting of bupropion 450 mg daily, buspirone 15 mg three times daily, gabapentin 600 mg three times daily, topiramate 100 mg twice daily, venlafaxine 300 mg daily, and pramipexole 3 mg three times daily. Comprehensive chart review revealed that pramipexole 0.5 mg three times daily was initiated in June 2019 for the treatment of RLS; unfortunately, clinical documentation did not provide a clear description of the symptoms which prompted this diagnosis. Laboratory workup was not completed at this time. Due to an incomplete response and worsening RLS symptoms, described by the patient as feeling intense discomfort in her legs at night and only relieved by movement significantly disturbing her sleep, the pramipexole dose was progressively escalated, reaching 1.5 mg three times daily by December 2019 and ultimately 3 mg three times daily by September 2024. IDA was first documented in September 2021 with TSAT of 5%, serum iron of 20 mcg/dl, and TIBC of 412 mcg/dL; ferritin was not assessed. She was not receiving iron supplementation prior to hospitalization.

Within 2 months following initiation of pramipexole, the patient developed severe orofacial pain accompanied by skin lesions, with histopathological analysis suggesting prurigo nodularis. Shortly thereafter, she underwent a series of dental interventions which failed to alleviate her pain. In the psychiatric hospital, a surgical consultation was ordered which excluded otolaryngological or dental pathology. However, the consultation confirmed the presence of self-induced perioral excoriations, noting symptomatic improvement with tactile manipulation, consistent with the patient’s reported compulsive “unroofing” behavior.

During the patient’s spring 2025 psychiatric hospitalization, she continued to report severely depressed mood and suicidal ideation. Further psychiatric evaluations revealed tactile hallucinations of infestation around the mouth that had emerged shortly after pramipexole initiation, driving the compulsive excoriation behavior and occasional auditory hallucinations. The supratherapeutic dosing of pramipexole and the clear temporal relationship with tactile hallucination onset led to the clinical hypothesis that these perceptual disturbances represented medication-induced adverse effects. Furthermore, her impulsive behavioral pattern resulting in engagement with fraudulent financial schemes raised clinical suspicion for an iatrogenic cause. Based on this clinical formulation, the decision was made to initiate a gradual pramipexole taper with the goal of complete discontinuation.

Pramipexole was slowly tapered over 4 weeks and discontinued while maintaining gabapentin for RLS. Concurrently, the patient received 10 sessions of electroconvulsive therapy and received iron supplementation for her IDA. Following completion of this treatment plan, both auditory and tactile hallucinations resolved, the perioral lesions healed completely and RLS remained controlled without additional medications. The depressive episode remitted, and suicidal ideation resolved. The patient further developed insight regarding her vulnerability to financial exploitation, recognizing these behaviors as distinctly uncharacteristic of her baseline judgment and decision-making patterns. She was able to be discharged from the psychiatric hospital with local follow-up.

## 3 Discussion

Dopamine is synthesized endogenously from the amino acid L-tyrosine via tyrosine hydroxylase and L-DOPA decarboxylase enzymes and can be further metabolized into norepinephrine and epinephrine, which, together with dopamine, are collectively referred to as catecholamines. In the central nervous system, dopamine is produced by presynaptic neurons in neural pathways and plays a key role in affective, executive, and motivation processes (mesolimbic and mesocortical tracts), the regulation of pituitary hormones (tuberoinfundibular tract), and control of motor movements (nigrostriatal tract) (Kandel et al., 2013). Dysfunction in dopamine signaling has been implicated in a wide range of clinical syndromes including psychosis, compulsive behaviors, endocrine disorders, and movement disorders such as Parkinson’s disease and RLS.

RLS, also known as Willis–Ekbom disease, is a neurologic sensorimotor disease which is clinically diagnosed based primarily on patient-reported symptoms as there is no objective testing for the condition. The essential features of RLS are: (1) an urge to move one or both lower extremities, usually accompanied by dysesthesia, which (2) begins or worsens with physical inactivity, (3) is relieved by movement, (4) occurs primarily at night, and (5) is not better explained by another medical condition (Allen et al., 2014). Prevalence is estimated at 2.5% of the population with increased morbidity and mortality compared to the general population (Winkelman, 2021).

While the pathophysiology of RLS remains poorly understood, recent evidence suggests a complex interplay between genetic predisposition, brain iron deficiency, and dysregulation of the dopaminergic system (Manconi et al., 2021). Brain iron deficiency is the most consistently implicated risk factor in the pathophysiology of RLS. This appears to be related to impaired iron transport or regulation within the central nervous system, particularly in the substantia nigra and thalamus, which subsequently disrupts normal dopaminergic neurotransmission (Ferré et al., 2019). Serum iron studies should be routinely obtained in all patients with RLS with a serum ferritin threshold of <30 ng/mL having a 98% specificity and 92% sensitivity for absolute iron deficiency. As ferritin is an acute phase reactant and is elevated during periods of inflammation, a TSAT threshold <20% may also be used to assess for iron deficiency during comorbid inflammatory states (Auerbach et al., 2025). Treatment with intravenous iron supplementation is recommended when ferritin levels are <100 ng/mL (Winkelman et al., 2025).

The dopaminergic system also plays a central role in the pathophysiology of RLS. Evidence indicates abnormal central dopaminergic signaling, particularly within the nigrostriatal and mesolimbic tracts (Khan et al., 2017). Given the role of dopamine dysregulation, DAs have a long history of therapeutic use in RLS and related conditions such as Parkinson’s disease. These medications directly stimulate dopamine receptors, mimicking the effects of endogenous dopamine. The first generation of DAs was derived from the ergot fungi and includes cabergoline, bromocriptine, and pergolide. However, this class of medications has been associated with increased risk of valvular heart disease (Schade et al., 2007) and is no longer considered a first line option for either Parkinson’s disease or RLS but is still used in the treatment of certain endocrine disorders. Subsequent, non-ergot DAs have a much lower risk of valvular disease (Mu et al., 2025) and the United States Food and Drug Administration (FDA) has approved three non-ergot DAs for the treatment of RLS: ropinirole (maximum dose 4.0 mg), pramipexole (0.5 mg), and rotigotine (3.0 mg) (Winkelman, 2021). Until recently, both ropinirole and pramipexole were considered standard interventions, with the AASM rating their use as “benefits clearly outweigh harms” based on high-quality evidence (Aurora et al., 2012) and the American Academy of Neurology recommending both pramipexole and rotigotine with the highest evidence level (Winkelman et al., 2016).

Unfortunately, in the past decade, accumulating evidence of several adverse effects has cast DAs in a less favorable light. The most well recognized is *augmentation*, a paradoxical worsening of RLS symptoms over time with continued DA use, characterized by earlier daily onset, greater intensity, and extension to other body regions (Romero-Peralta et al., 2020). In a community-based sample, rates of augmentation were 24% with ropinirole and 11% with pramipexole (Allen et al., 2011). To counteract this phenomenon, both patients and clinicians - each often unaware of the underlying process - may enter a Sisyphean cycle of escalating doses. This approach provides transient relief but ultimately exacerbates symptoms. We suspect this mechanism contributed to our patient’s clinical course, as her DA dose was progressively titrated to 18 times the FDA-recommended maximum (a dosage we initially assumed was a documentation error), yet she continued to report severe and refractory RLS symptoms.

Another important adverse effect of long-term DA therapy is the emergence of *impulse control disorders* (ICDs). ICDs are characterized by the iatrogenic development of compulsive behaviors such as pathologic gambling, hypersexuality, compulsive shopping, and binge eating, with a strong association reported for both pramipexole and ropinirole (Moore et al., 2014). These effects are thought to be related to the drug’s preferential D_3_ receptor agonism, as D_3_ receptor antagonists have shown promise in the treatment of substance use disorders (Newman et al., 2012). In our case, the onset of uncharacteristic and poorly planned financial speculation and romantic interactions raised concern for a DA-induced ICD.

However, the patient’s most distressing symptom, which she identified as the primary contributor to her suicidal ideation and reason for presenting to the emergency department, was severe orofacial pain. Despite multiple evaluations by community medical and dental professionals and subsequent referral to otolaryngology during hospitalization, no clear structural etiology for her pain was identified. We suspect she was experiencing tactile hallucinations accompanied by a delusional belief of infestation, which likely drove compulsive excoriation. Tactile hallucinations are most commonly associated with recreational drugs that increase synaptic dopamine levels (McKetin et al., 2017), but have also been reported with pramipexole (Nakamura and Koo, 2016) and other non-ergot DAs (Mu et al., 2025). Because the onset of these psychotic symptoms occurred shortly after initiation of pramipexole, we believe they were also attributable to DA therapy. Given that her severe mood symptoms ultimately required electroconvulsive therapy while she was undergoing a taper of the DA, it remains unclear whether these psychotic features would have fully remitted with discontinuation of the offending agent alone.

Growing recognition of the adverse effects associated with DAs has led to reversals in professional guidelines. In its most recent update to the 2012 recommendations, released earlier this year, AASM now conditionally advises against the routine use of pramipexole, ropinirole, or transdermal rotigotine for adults with RLS, citing moderate- to low-certainty evidence. These agents may still be considered in patients who place a higher value on short-term symptom relief and are willing to accept the increased risk of long-term adverse effects, particularly augmentation. Gabapentinoids now carry a strong recommendation for use with a moderate certainty of evidence. Iron supplementation remains advised for patients with iron deficiencies (Winkelman et al., 2025).

In summary, we present the case of a 64-year-old woman who developed severe RLS and later experienced iatrogenic harm from suboptimal management and adverse effects of DA therapy. These effects led to severe depression and suicidal ideation that ultimately required psychiatric hospitalization. Initial evaluation of RLS should include serum iron studies to assess for IDA, and iron should be repleted if deficient. The patient’s RLS symptoms improved markedly after iron supplementation and normalization of ferritin and TSAT even while RLS-specific interventions were discontinued. Earlier treatment of her IDA may have prevented later complications. At the time of diagnosis, a non-ergot DA was considered first-line therapy for RLS, however current guidelines now recommend against this class of medications due to risks of augmentation, ICDs, and psychosis. The patient’s gabapentin therapy, now regarded as a first-line option, was continued. Many clinicians and patients may not yet be aware of these updates given their recent publication. We hope this case report and accompanying review of the literature will help highlight these important changes and their clinical rationale.

## 4 Patient perspective

The patient reported substantial improvement in quality-of-life following treatment intervention, with particular emphasis on relief from distressing tactile perceptual disturbances and restoration of financial judgment and decision-making capacity. Written informed consent was obtained from the patient for publication of this case report in accordance with institutional guidelines and ethical standards.

## Data Availability

The original contributions presented in the study are included in the article/supplementary material, further inquiries can be directed to the corresponding author.
